# Solo Act or Teamwork: Contrasting Characteristics of Caregiving Networks by Dementia Status and Single Caregiver Structures

**DOI:** 10.1002/alz70858_097452

**Published:** 2025-12-24

**Authors:** Amanda N Leggett, Shushun N Ren, Sophia Tsuker, Wen‐hua Lai, Natasha L Nemmers

**Affiliations:** ^1^ Wayne State University, Detroit, MI, USA; ^2^ Wayne State University Institute of Gerontology, Detroit, MI, USA

## Abstract

**Background:**

Research on family caregiving for individuals with dementia has primarily focused on a single, “primary” caregiver (e.g., adult child, spouse), while neglecting broader networks of care. To understand how and why caregiving networks form, it is important to consider 1) how characteristics of individuals with dementia and their caregiver(s) differ by whether they have one (single caregiver) versus two or more caregivers (caregiving network) and 2) how characteristics of caregiving networks vary by dementia status.

**Method:**

Using the 2022 National Health and Aging Trends Study, a nationally representative sample of Medicare‐eligible adults, and the associated National Study of Caregiving, we compare demographics, network characteristics, and caregiver well‐being (e.g., emotional difficulty of care, caregiving overload) across 1) individuals without dementia (*n* = 726) versus individuals with dementia (*n* = 479), and 2) individuals with dementia who have one caregiver (*n* = 129) versus those who have two or more caregivers (*n* = 350). T‐tests and Chi‐square tests are run to compare mean differences between groups.

**Result:**

Relative to individuals without dementia, individuals with dementia had significantly more female caregivers, larger networks, task sharing among caregivers, and more immediate family members but fewer non‐family members in their networks. Caregivers in dementia networks also reported significantly greater emotional difficulty, family disagreement over care, overload, and worse relationship quality, but more caregiving gains. Compared to individuals with one caregiver, individuals with dementia with a caregiving network were significantly more likely to be female, Black, less educated, and have more self‐care needs. Caregiving networks were less likely to include a spouse but more likely to include a daughter, immediate family member, and non‐family member. Solo caregivers reported significantly greater emotional and physical difficulty of care than those in a caregiving network.

**Conclusion:**

Relative to individuals with one caregiver, dementia caregiving networks are more likely to form around diverse individuals with greater care needs, and these networks may diffuse stress for caregivers. Furthermore, networks of individuals with dementia, relative to older adults without dementia, are larger and engaging in significantly more collaborative care. Interventions and services for caregivers should incorporate the needs of non‐traditional caregivers and broader networks of care.

